# Supplementary material for: Nicotine exacerbates MASH via inducing intestinal dysbiosis and barrier dysfunction
Source: Commun Biol. 2025 Dec 5;8:1748. doi: 10.1038/s42003-025-09280-5 (PMC12680697; doi:10.1038/s42003-025-09280-5)
Supplement: Supplementary file 2 — Supplementary Information [file 42003_2025_9280_MOESM2_ESM.pdf]

Supplementary Table 1.

Gene	Forward (5′-3′)	Reverse (5′-3′)
β-actin	CAT TGC TGA CAG GAT GCA GAA GG	TGC TGG AAG GTG GAC AGT GAG G
ZO-1	GGG CCA TCT CAA CTC CTG TA	AGA AGG GCT GAC GGG TAA AT
Occludin	ACT ATG CGG AAA GAG TTG ACA G	GTC ATC CAC ACT CAA GGT CAG
Claudin-1	GGG TTT CAT CCT GGC TTC TCT G	CTG AGC GGT CAC GAT GTT GTC
HIF-1A	CAA GAT CTC GGC GAA GCA A	GGT GAG CCT CAT AAC AGA AGC TTT
IL-1β	TTC ATC TTT GAA GAA GAG CCC AT	TCG GAG CCT GTA GTG CAG TT
IL-6	TGG AAA TGA GAA AAG AGT TGT GC	CCA GTT TGG TAG CAT CCA TCA
TNFA	ACG CTC TTC TGT CTA CTG AAC TTC	GGT TTG TGA GTG TGA GGG TCT G
IL-10	GGA CAA CAT ACT GCT AAC CGA CTC	TGG ATC ATT TCC GAT AAG GCT TGG

Supplementary Table 1. Primer sequences for qRT-PCR.

Supplementary Fig. 1.

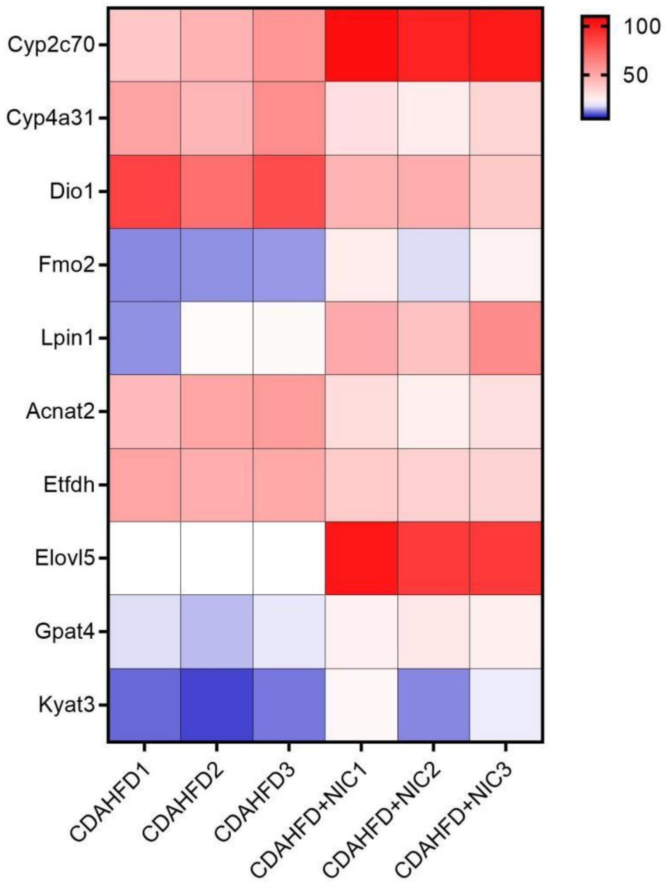

Supplementary Fig. 1. Liver gene expression in nicotine-exposed MASH mice. a, Heatmap of DEGs related to metabolism between mice with and without nicotine treatment when fed an CDAHFD. DEGs, differentially expressed genes.

Supplementary Fig. 2.

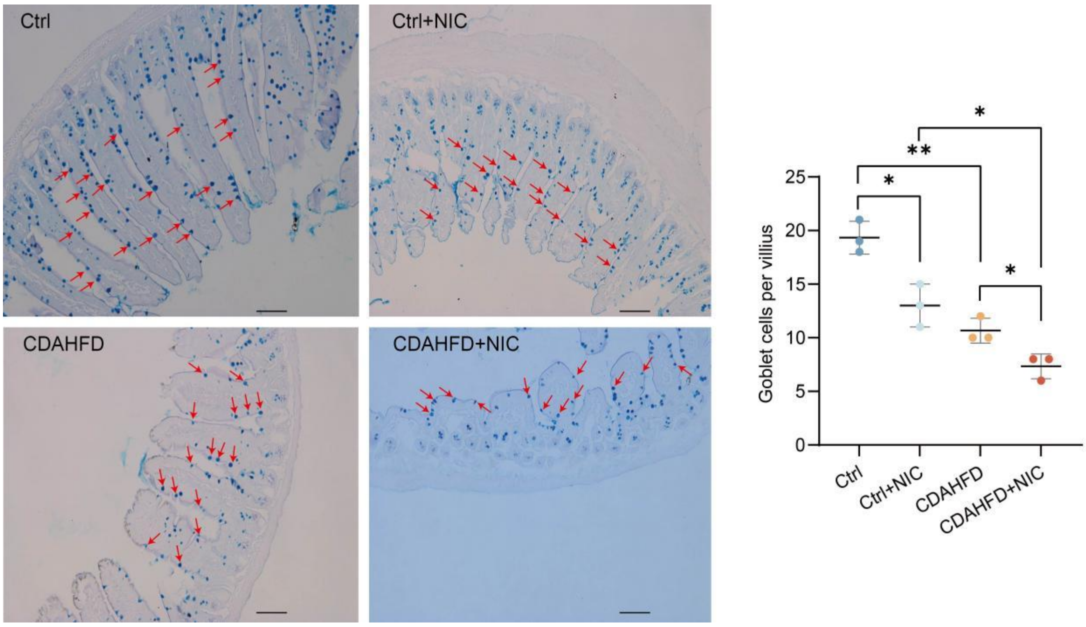

Supplementary Fig. 2. Goblet cell staining and quantification of the ileal tissues. Arrows denote Goblet cells. Scale bar = 200 μm. *$P < 0.05$, **$P < 0.01$. Data are presented as mean ± SD; n=3.

Supplementary Fig. 3.

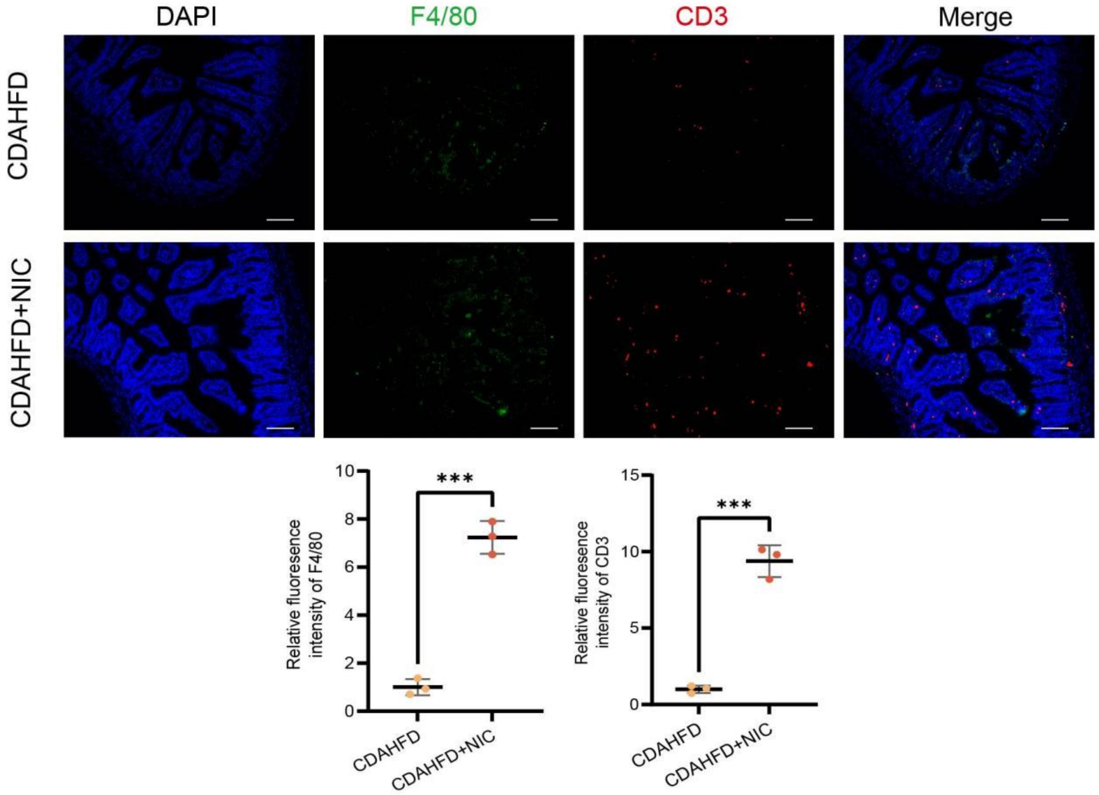

Supplementary Fig. 3. F4/80 and CD3 staining of the ileal tissues. Scale bar = 200 μm. ****P* < 0.001. Data are presented as mean ± SD; n=3.

Supplementary Fig. 4.

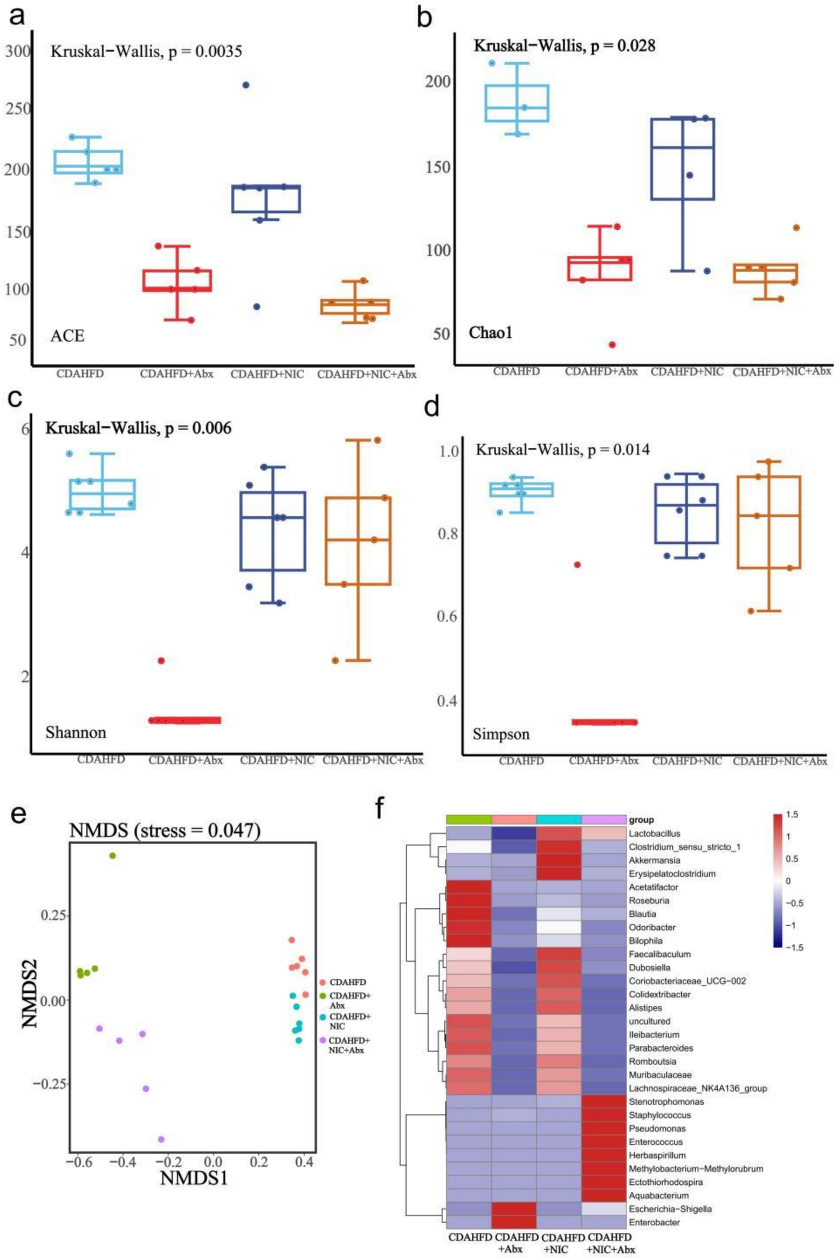

Supplementary Fig. 4. Abx treatment effectively depleted fecal microbiota. a-d, alpha diversity results. **e,** beta diversity with PCoA. **f,** Heatmap of bacteria at the genus level. Data are presented as mean ± SD; n=6.

Supplementary Fig. 5.

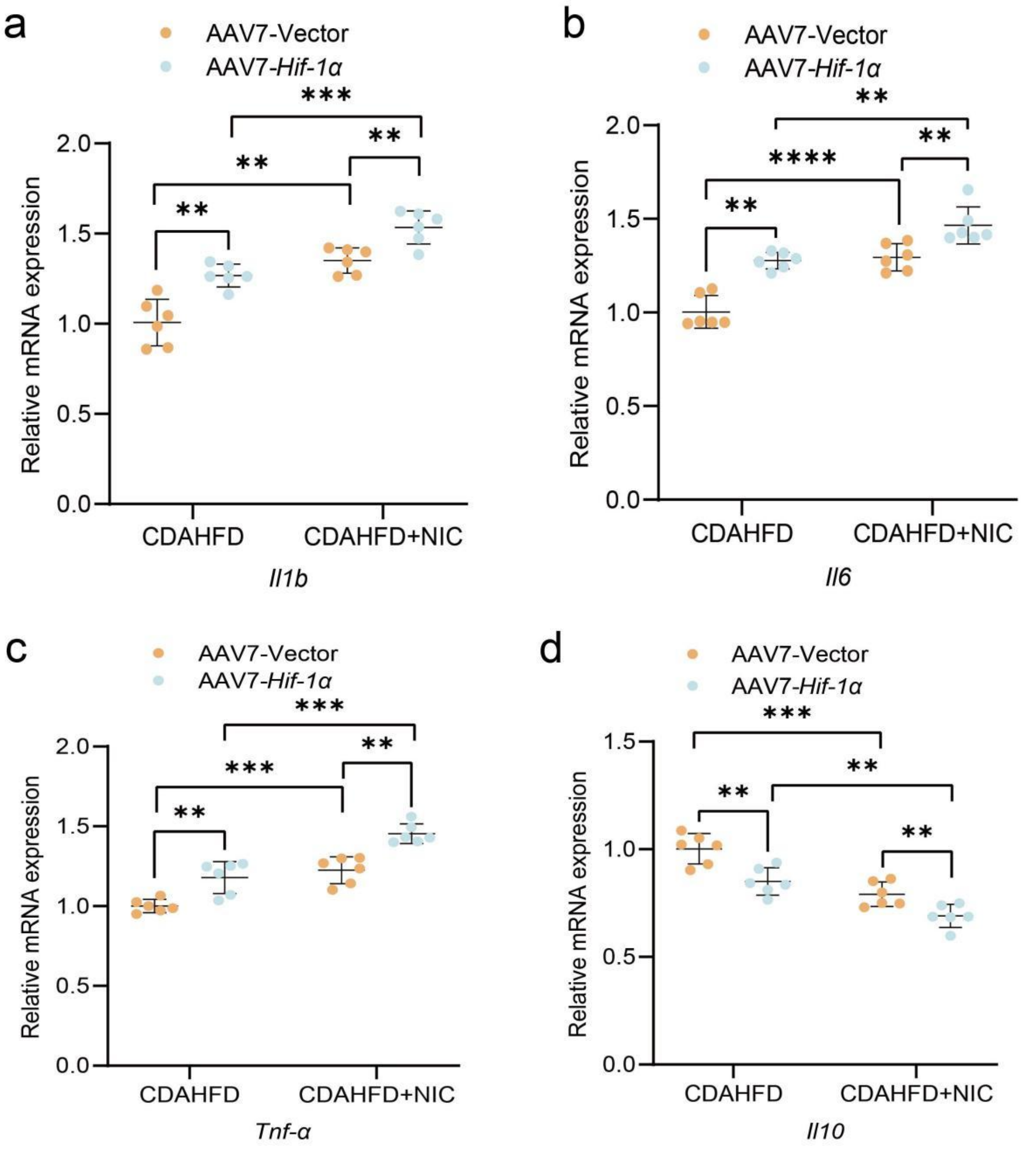

Supplementary Fig. 5. Intestinal gene expression of inflammatory cytokines. a-c, Pro-inflammatory cytokines (*Il1b*, *Il6*, *Tnf-α*). **d,** Anti-inflammatory cytokines (*Il10*). $^{**}P < 0.01$, $^{***}P < 0.001$. Data are presented as mean ± SD; n=6.

Supplementary Fig. 6.

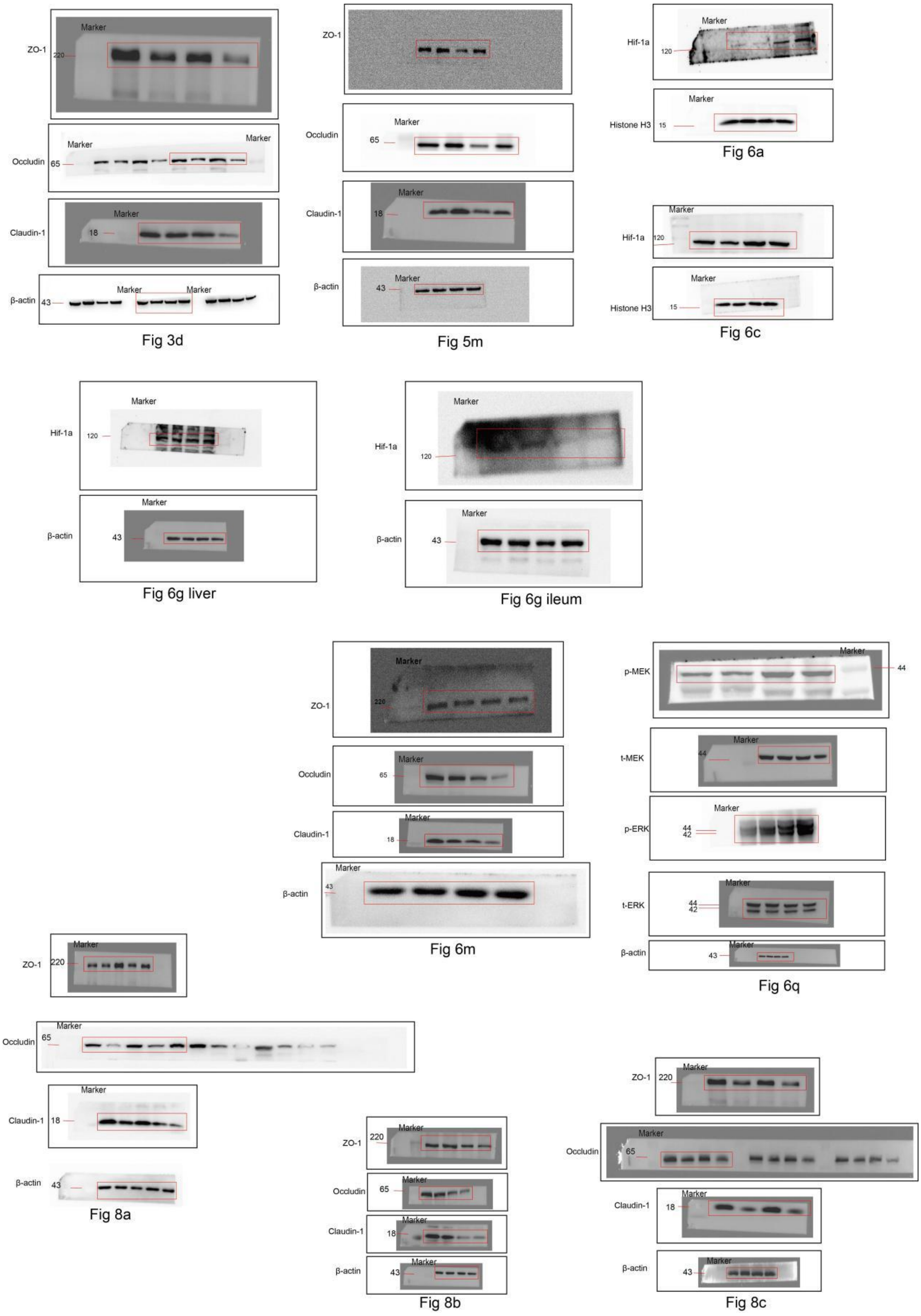

Supplementary Fig. 6. Uncropped/unedited images of blots.
